# Gut Microbial Diversity Assessment of Indian Type-2-Diabetics Reveals Alterations in Eubacteria, Archaea, and Eukaryotes

**DOI:** 10.3389/fmicb.2017.00214

**Published:** 2017-02-14

**Authors:** Shrikant S. Bhute, Mangesh V. Suryavanshi, Suyog M. Joshi, Chittaranjan S. Yajnik, Yogesh S. Shouche, Saroj S. Ghaskadbi

**Affiliations:** ^1^Department of Zoology, Savitribai Phule Pune UniversityPune, India; ^2^Microbial Culture Collection-National Centre for Cell SciencePune, India; ^3^Diabetes Unit, KEM Hospital and Research CentrePune, India

**Keywords:** diabetes, gut microbiota, eubacteria, archaea, eukarya, amplicon sequencing

## Abstract

Diabetes in India has distinct genetic, nutritional, developmental and socio-economic aspects; owing to the fact that changes in gut microbiota are associated with diabetes, we employed semiconductor-based sequencing to characterize gut microbiota of diabetic subjects from this region. We suggest consolidated dysbiosis of eubacterial, archaeal and eukaryotic components in the gut microbiota of newly diagnosed (New-DMs) and long-standing diabetic subjects (Known-DMs) compared to healthy subjects (NGTs). Increased abundance of phylum Firmicutes (*p* = 0.010) and Operational Taxonomic Units (OTUs) of *Lactobacillus* (*p* < 0.01) were observed in Known-DMs subjects along with the concomitant graded decrease in butyrate-producing bacterial families like Ruminococcaceae and Lachnospiraceae. Eukaryotes and fungi were the least affected components in these subjects but archaea, except *Methanobrevibacter* were significantly decreased in them. The two dominant archaea viz. *Methanobrevibacater* and *Methanosphaera* followed opposite trends in abundance from NGTs to Known-DMs subjects. There was a substantial reduction in eubacteria, with a noticeable decrease in Bacteroidetes phylum (*p* = 0.098) and an increased abundance of fungi in New-DMs subjects. Likewise, opportunistic fungal pathogens such as *Aspergillus, Candida* were found to be enriched in New-DMs subjects. Analysis of eubacterial interaction network revealed disease-state specific patterns of ecological interactions, suggesting the distinct behavior of individual components of eubacteria in response to the disease. PERMANOVA test indicated that the eubacterial component was associated with diabetes-related risk factors like high triglyceride (*p* = 0.05), low HDL (*p* = 0.03), and waist-to-hip ratio (*p* = 0.02). Metagenomic imputation of eubacteria depict deficiencies of various essential functions such as carbohydrate metabolism, amino acid metabolism etc. in New-DMs subjects. Results presented here shows that in diabetes, microbial dysbiosis may not be just limited to eubacteria. Due to the inter-linked metabolic interactions among the eubacteria, archaea and eukarya in the gut, it may extend into other two domains leading to trans-domain dysbiosis in microbiota. Our results thus contribute to and expand the identification of biomarkers in diabetes.

## Introduction

The eubacterial assemblage associated with the human body together with other microbes like archaea, eukaryotes and fungi are referred to as “microbiota.” Trillions of these microbes that live in our distal gut are believed to be co-evolving with their hosts (Ley et al., 2008). Within the gut, microbes interact amongst themselves and their host; together, their metagenomes contain genes that act as a repertoire of metabolic functions which influence human health (Clemente et al., 2012). Recent studies have revealed that the gut microbiota is subjected to variations in the host's diet (Turnbaugh et al., 2009), genotype (Spor et al., 2011) and health status (Cénit et al., 2014). Any perturbation in the delicate balance between microbial consortia and host results in “dysbiosis,” sometimes leading to severe ailments in the host. Thus, gastrointestinal disorders such as inflammatory bowel disease (Frank et al., 2007) and colitis (Lucke et al., 2006), as well as metabolic disorders such as obesity (Turnbaugh et al., 2006) and diabetes (Qin et al., 2012; Karlsson et al., 2013; Zhang et al., 2013) are found to be associated with the distinct pattern of gut microbiota in which certain OTUs/species are present in different proportions.

Although studies on gut microbiota are largely dominated by eubacteria, in recent years, studies on gut-inhabiting archaea, (Scanlan et al., 2008; Gaci et al., 2014) fungi (Dollive et al., 2012; Wang et al., 2014) and eukaryotes (Pandey et al., 2012; Grattepanche et al., 2014) are being conducted to understand their distribution and possible role in human health. Thus, archaea such as genus *Methanobrevibacter* has been linked with human diseases like obesity (Million et al., 2012) and periodontitis (Lepp et al., 2004). Fungi residing in the gut are associated with diseases such as colorectal adenomas (Luan et al., 2015) and, Crohn's disease (Li Q. et al., 2014). Similarly, eukaryotes in the gut are found to be very complex and correlated with human diseases (Gouba et al., 2014). Thus, besides the fact that reports on gut archaea, fungi and eukaryotes are lagging, studies such as these are a clear indication that these microbes together with eubacteria forms a very complex ecosystem in the gut and their functional role in human health and diseases needs to be evaluated thoroughly.

Studies conducted in Indian population suggest compositional differences in gut microbiota and how it differs from the western population (Patil et al., 2012; Bhute et al., 2016). Therefore, considering the unique gut microbial features of Indian population (Bhute et al., 2016) efforts to define extents of perturbation in gut microbial communities of diabetic subjects from India will help us to decipher the association between gut microbial composition and diabetes. India is one of the global capitals of diabetes with an estimated 69.1 million diabetic patients in the year 2015 (International Diabetes Federation, 2015). The explosive epidemic of diabetes in India is incompletely explained, although various contributing factors are suggested. Compared to diabetic patients in the western world, Indian diabetic patients have unique and paradoxical characteristics. These include possible heightened genetic predisposition (Ramachandran et al., 2012), intrauterine undernutrition (thrifty phenotype) leading to epigenetic predisposition (Yajnik, 2001), the manifestation of diabetes at an earlier age and at a much lower body mass index (BMI) compared to white Caucasians (Yajnik, 2004). Diabetes seems to be precipitated in this population by rapid economic and nutritional transition and rural-urban migrations (Anjana et al., 2011).

Based on above facts, we hypothesized that the dysbiosis in gut microbiota may not be limited to just eubacteria but other two domains (Archaea and Eukarya) too are disturbed due to the disease condition or vice-versa. In the present study, we investigated the composition of the intestinal microbiota of newly diagnosed (New-DMs) and long-standing diabetic subjects (Known-DMs) and compared it with normal glucose tolerant subjects (NGTs). We used Ion torrent PGM sequencing technology, to analyse eubacterial and archaeal 16S rRNA gene, 18S rRNA gene from eukaryotes and fungal ITS tagged amplicon from fecal samples.

## Materials and methods

### Participants and sample collection

We studied 49 adults, who are parents of children in the Pune Children Study (PCS) conducted by Diabetes Unit of KEM Hospital Research Centre (Yajnik et al., 1995). They have been followed up since 1995 along with their children with serial glucose tolerance testing. The present study refers to clinical and metabolic follow-up in 2009. The study and the experimental protocols followed were approved by Ethics Committee of KEM Hospital Research Centre, Pune, India (Study number 0,847), and separate written informed consent was obtained from each participant. Inclusion criteria in NGTs group was the absence of any apparent acute or chronic disorders. New-DMs were the participants that were diagnosed with type 2 diabetes during the routine check-up, were not on anti-diabetic treatment until sample collection and free from any acute and chronic illness. Known-DMs subjects were known cases of type 2 diabetes in PCS cohort, were on anti-diabetic treatment at least for the past 1 year and free from any acute and chronic illness. General exclusion criteria for all three groups were subjects undergoing dietary intervention, use of antimicrobial in past 3 months and major surgeries of the gastrointestinal tract. All participants were admitted to Diabetes Unit the evening before the investigations. Anthropometry was measured by trained observers according to standard protocols. The following morning, fasting blood specimens were assessed for plasma glucose, insulin and lipids. Sixteen known diabetic subjects underwent only fasting and post-breakfast glucose measurements. In the remaining subjects, an oral glucose tolerance test (75 g anhydrous glucose) was carried out according to the (Alberti and Zimmet, 1998) protocol. Fecal samples were collected from all participants in a sterile container and preserved at −80°C until DNA extraction.

### Measurement of biochemical parameters

Plasma glucose, cholesterol, HDL-cholesterol, and triglyceride concentrations were measured using standard enzymatic methods (Hitachi 902, Germany). Between-batch coefficients of variation for all these assays were <3% in the normal range. Plasma insulin was measured using Delfia technique (Victor 2, Wallac, Turku, Finland). Overweight was defined as BMI ≥25 kg/m^2^ and <30 kg/m^2^, and obesity as BMI ≥30 kg/m^2^. Diabetes mellitus was diagnosed if fasting plasma glucose ≥126 mg/dl or 120-min plasma glucose ≥200 mg/dl. Hypercholesterolaemia was defined as plasma total cholesterol ≥200 mg/dl, hypertriglyceridaemia as plasma triglyceride concentration ≥150 mg/dl and low HDL-cholesterol as HDL-cholesterol concentration <40 mg/dl for men and <50 mg/dl for women. Hypertension was defined as systolic blood pressure (SBP) ≥130 mmHg or diastolic blood pressure (DBP) ≥85 mmHg.

### Sequencing of 16S rRNA gene amplicons

Total community DNA was extracted from each fecal sample using QIAmp DNA Stool Mini kit (Qiagen, Madison USA) as per manufacturer's protocol. The PCR amplification and sequencing of resulting amplicons was performed as described earlier (Bhute et al., 2016). Briefly, the concentration of extracted DNA was measured using Nanodrop-1000, (Thermo Scientific, USA). DNA concentration was normalized to 100 ng/μl and used as a template for amplification of 16S rRNA gene. PCR was set up in 50 μl reaction using AmpliTaq Gold PCR Master Mix (Life Technologies, USA) and with 16S rRNA V3 region specific bacterial universal primers: 341F (5′-CCTACGGGAGGCAGCAG-3′) and 518R (5′-ATTACCGCGGCTGCTGG-3′) (Bartram et al., 2011). Following conditions were used for PCR: initial denaturation at 94°C for 4 min, followed by 20 cycles of 94°C for 1 min, 56°C for 30 s, and 72°C for 30 s with final extension at 72°C for 10 min. PCR products were purified using Agencourt AMPure XP DNA purification Bead (Beckman Coulter, USA). Resulting PCR products were end-repaired and ligated with sample-specific barcode adaptor as explained in Ion Xpress™ Plus gDNA Fragment Library Preparation user guide. Prior to sequencing, fragment size distribution and molar concentrations of amplicons were assessed on Bioanalyser 2,100 (Agilent Technologies, USA) using High Sensitivity DNA Analysis Kit. All amplicons were diluted to the lowest molar concentration and pooled into sets of 10 samples. Emulsion PCR was carried out on Ion OneTouchTM System using Ion OneTouch™ 200 Template Kit v2 DL (Life Technologies) as explained in Ion OneTouchTM 200 Template Kit v2 user manual. The resulting template-positive Ion Sphere particles were enriched using Ion OneTouch ES system and sequencing of amplicon libraries was carried out on 316 chips using Ion Torrent PGM system and Ion Sequencing 200 kit following the user guide: Ion PGM™ Sequencing 200 Kit v2.

### Sequencing of archaeal 16S, eukaryotic 18S and fungal ITS genes

The archaeal 16S, eukaryotic 18S and fungal ITS1 genes were PCR amplified using primers listed in Supplementary Table 1. The resulting PCR products were purified using Agencourt AMPure XP DNA purification Bead (Beckman Coulter, USA) and quantified using Nanodrop-1000 (Thermo Scientific, USA). Then, PCR products of all NGTs samples (*n* = 19), all New-DMs (*n* = 14) and all Known-DMs (*n* = 16) were pooled by mixing equal quantities of concentration normalized PCR products. This way we obtained three pools for each group, NGTs, New-DMs and Known-DMs for archaeal 16S rRNA, eukaryotic 18S rRNA and fungal ITS1 genes. All the pooled samples were then sequenced using Ion Torrent PGM. Since fungal ITS amplicons varied in length, we fragmented 100 ng of it with Ion Shear Enzyme mix (Ion Xpress Plus Fragment Library preparation kit, Life Technologies) for 20 min and 200 bp size fragments were selected before adapter ligation step (Tang et al., 2015).

### Sequence processing and bioinformatics analysis of eubacterial 16S rRNA gene amplicons

All PGM quality-approved reads from 49 samples were exported as sample specific fastq files and pre-processed in Mothur pipeline (Schloss et al., 2009) with following conditions: (1) minimum length–150 bp, (2) maximum length–200 bp, (3) maximum homopolymer–5, (4) maximum ambiguity–0, and (5) average quality score–20. This way we derived a total of 2.1 million high-quality amplicon reads from 49 samples; subsequently, these reads were pooled as single FASTA file for further analysis in QIIME: Quantitative Insights Into Microbial Ecology (Caporaso et al., 2010). Briefly, reads were binned into Operational Taxonomic Units (OTUs) at 97% sequence similarity using UCLUST algorithm and single sequence from each OTU was picked out for further analysis. The PyNAST algorithm was used to align representative sequences against Greengenes core set; all unaligned and chimeric sequences were excluded from alignment and downstream analysis. Then lane masking was applied to the alignment to retain conserved regions of 16S rRNA and a phylogenetic tree was inferred using FastTree 2.1.3. Additionally, all reads were assigned to the lowest possible taxonomic rank by utilizing RDP Classifier 2.2 with a confidence score of 80%. Alpha diversity measures such as Chao1 index (Chao, 1984) and Shannon index (Shannon, 1948) were inferred. Phylum level abundance data and alpha diversity indices were compared among the three groups using the non-parametric tests such as Wilcoxon sum rank test and Kruskal-Wallis rank sum test. To assess beta diversity among three study groups, we applied phylogenetic distance based UniFrac (both unweighted and weighted) analysis and the results are visualized as Principal coordinate plots. To determine differentially abundant OTUs among the three groups, OTU table was filtered such that at least 8 sample will have that OTU to be retained in the OTU table. Kruskal-Wallis rank sum test was then applied to filtered OTU table containing 1969 OTUs. We next applied supervised machine learning approach (Random Forest) to identify OTUs that were indicators of community differences in three groups. This was done by estimating the amount of error introduced if a particular OTU is removed from a group of indicator OTUs and assigning it an importance score.

### Clustering of samples into enterotypes

To understand whether disease state has any effect on the composition of enterotypes, we applied original measurements proposed by Arumugam et al. (2011) and as detailed at http://enterotyping.embl.de (Arumugam et al., 2014) to partition the samples into distinct enterotypes clusters. Briefly, the genus level abundance data was segregated according to the three categories, imported in R and clustered using partitioning around medioid (PAM) algorithm followed by determination of optimal number of clusters by utilizing Calinski-Harabasz (CH) index. Finally, results of between class (BC) analyses were visualized as principal component analysis. Additionally, taxa that influenced partitioning of samples into enterotypes (drivers of enterotype) were identified based on their abundance in a particular enterotype.

### Bioinformatics analysis of archaeal 16S, eukaryotic 18S and fungal ITS genes

Most of the steps for analysis of pooled archaeal 16S, eukaryotic 18S and fungal ITS1 genes were similar as that of eubacterial 16S rRNA gene, except for the fact that QIIME compatible SILVA_111 database (Quast et al., 2013) for archaeal 16S and eukaryotic 18S amplicons and QIIME compatible UNITE_12_11 database (Kõljalg et al., 2013) for fungal ITS amplicon was used during the OTU picking step.

### Prediction of ecological relationships

To predict ecological relationships among gut microbiota, microbial association network showing co-occurrence and co-exclusion pattern was built as described before (Faust et al., 2012). Briefly, genus level abundance data was imported to CoNet plugin (version 1.0.4 beta) in Cytoscape 3.0.0 environment (Shannon et al., 2003). To produce association network, 100 top and bottom edges were used with two measures of similarity (Pearson and Spearman) and three measures of dissimilarity (Bray-Curtis, Hellinger, and Kullback-Leibler). Spurious correlations due to compositional structure of relative abundances were avoided by bootstrapping and re-normalization and resulting networks were combined using Simes method followed by Benjamini-Hochberg-Yekutieli false discovery rate (FDR) correction with FDR cut-off of 0.05. Finally, all unstable edges outside the 95% confidence interval of bootstrap distribution score were removed and network was visualized and suitably edited.

### Metagenomic imputation

For metagenomic imputation, amplicon sequences were binned into OTUs at 97% similarity using closed-reference OTU picking in QIIME. The resulting OTU table was filtered such that at least 8 samples will have that OTU to retain it in OTU table. Resulting OTU table was then analyzed using online tool PICRUSt (Langille et al., 2013) at http://huttenhower.sph.harvard.edu/galaxy/. PICRUSt (phylogenetic investigation of communities by reconstruction of unobserved states) is a computational tool that uses marker gene data for prediction of functional composition of metagenome. Briefly, OTU abundance table was first normalized for 16S rRNA copy number against known gene copy number for each OTU. Functional predictions were categorized into KEGG pathways and an annotated table of predicted gene family counts (KOs) for each sample using predict metagenome option. Gene family table then categorized by function and further statistical analysis was performed in STAMP v2.0.1 (Parks and Beiko, 2010).

### Validation of eubacterial amplicon library results

Owing to their high precision, quantitative PCR-based assays were performed using group-specific primers to validate the major findings of eubacterial disturbances. Absolute quantification of total bacteria, phylum Bacteroidetes and genus *Lactobacillus* was performed using SYBR green master mix (Applied biosystems Inc. USA) on 7,300 Real-time PCR system from Applied Biosystems Inc. (USA). The primers and PCR conditions used for qPCR assays were described earlier (Suryavanshi et al., 2016). The findings of qPCR results were analyzed using Kruskal-Wallis test, bacterial groups with a *p*-value less than 0.05 were considered as significantly different among the three groups.

### Additional statistical analysis

Biochemical and anthropometric parameters were expressed as mean (SD) and ANOVA test is used to compare differences among the study groups. Different type of data generated through QIIME was imported and analyzed in ade4, vegan and ggplot2 packages within R software (R Core Team, 2013) environment. In addition, the relationship between biochemical parameters and microbiota were assessed using PERMANOVA: permutational multivariate analysis of variance test (Anderson and Walsh, 2013). Covariance between biochemical parameters dataset and genus abundance dataset was performed by using co-inertia analysis (Dray et al., 2003), these two datasets were connected to each other owing to the presence of same subjects.

### Availability of data

Raw sequences generated in the present study are deposited to NCBI SRA under accession number SRP041693.

## Results

### Summary of biochemical parameters

Biochemical and anthropometric characteristics are shown in Table 1. Out of the 49 participants, 19 were NGTs, 14 were New-DMs and 16 were Known-DMs. In the total study group, 8 participants were obese and 28 were overweight. Twelve participants had hypercholesterolemia, 16 had hypertriglyceridemia, 45 had low HDL and 8 were hypertensive.

**Table 1:** **Biochemical and Anthropometric parameters of the three study groups (Shown in the table mean ± SD)**.

	**NGTs**	**New-DMs**	**Known-DMs**
N	19	14	16
Age	48.85 ± 5.4	48.64 ± 5.68	50.62 ± 3.49
BMI kg/m^2^	25.52 ± 4.0	28.32 ± 2.58^a^	27.41 ± 3.53
Waist-hip ratio	0.92 ± 0.088	0.99 ± 0.071	0.96 ± 0.061
% body fat	35.68 ± 8.21	37.50 ± 6.12	35.46 ± 8.77
Fasting glucose mg/dl	93.8 ± 8.16	138.07 ± 47.35^a^	146.81 ± 44.90^b^
120 min glucose mg/dl	110.50 (18.40)	250.86 ± 77.76^a^	NA
PP glucose mg/dl	NA	NA	226.12 ± 58.43
Fasting insulin IU/L	9.16 ± 5.69	12.06 ± 6.11	10.94 ± 8.31
120 min insulin IU/L	71.39 ± 36.60	127.75 ± 183.76	NA
Systolic BP mmHg	115.66 ± 12.77	114.07 ± 37.81	110.69 ± 31.64
Diastolic BP mmHg	73.53 ± 10.74	73.43 ± 23.31	70.22 ± 20.34
Cholesterol mg/dl	166.63 ± 24.06	194.57 ± 44.15^a^	174.19 ± 38.11
Triglycerides mg/dl	120.60 ± 58	126.64 ± 54.41	137.18 ± 63.18
HDL cholesterol mg/dl	38.50 ± 8.15	40.79 ± 7.51	41.06 ± 7.76

a *p < 0.01 for New-DMs vs. NGTs*.

b *p < 0.01 for Known-DMs vs. NGTs*.

### Altered eubacterial diversity and OTU composition of diabetic subjects

**Figure 1:**
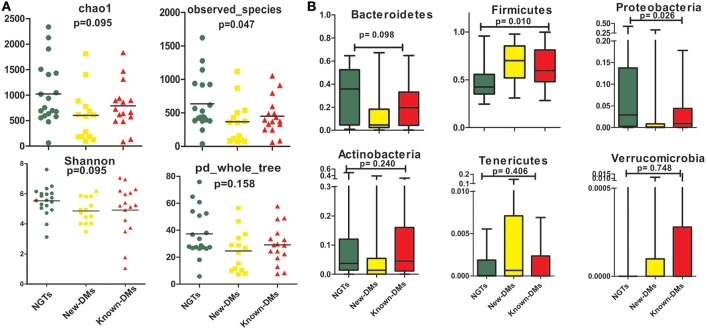
**Summary of diversity measurements. (A)** Assessment of alpha diversity indices in NGTs, New-DMs, and Known-DMs subjects. **(B)** Variation in phylum level abundance, the box depicts interquartile range between first and third quartiles and the line within box denotes median.

**Figure 2:**
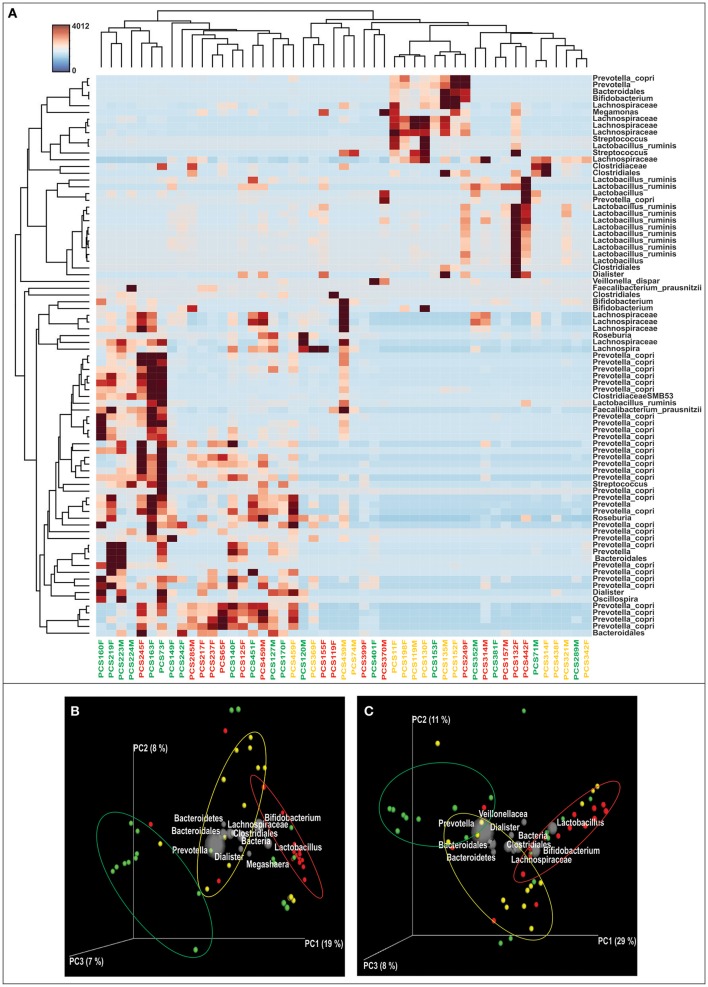
**Differentially abundant OTUs & Beta diversity analysis. (A)** Heatmap of the differentially abundant OTUs in three study groups as determined by Kruskal-Wallis test, subjects are identified as—green: NGTs, Yellow: New-DMs, and Red: Known-DMs. **(B)** Unweighted UniFrac distance based and **(C)**. Weighted UniFrac distance based PCoA bi-plots; the gray colored sphere represent a taxonomic group that influence clustering of samples (NGTs: green, New-DMs: yellow and Known-DMs: red) in particular area of the PCoA plot and its size demonstrate abundance of that taxonomic group.

**Figure 3:**
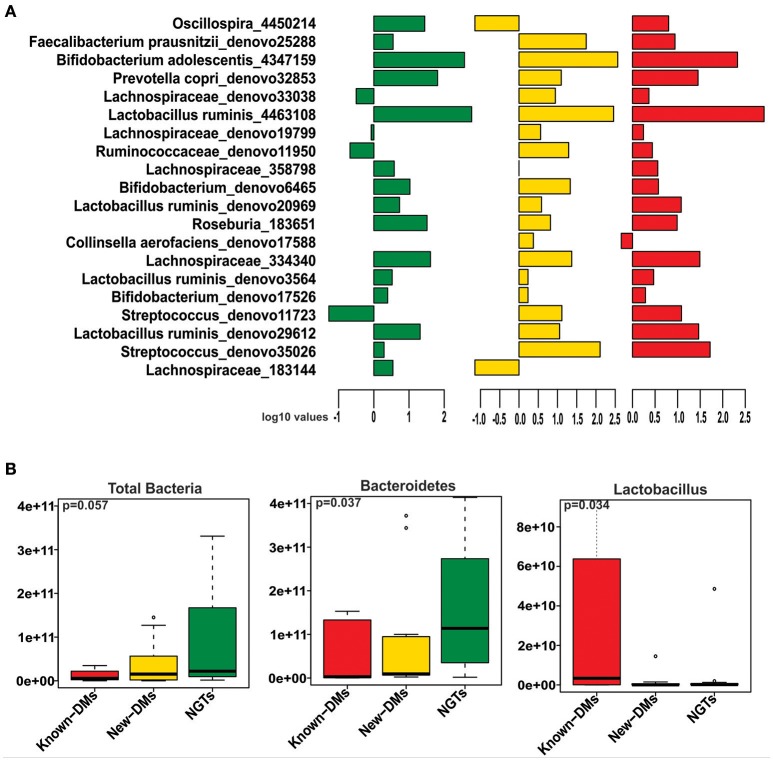
**(A)** Mean abundance of 20 discriminative OTUs as derived from Random Forest analysis (Green: NGTs, Yellow: New-DMs, Red: Known-DMs subjects). **(B)** Boxplot representing the absolute counts of total bacteria, phylum Bacteroidetes and genus *Lactobacillus* among NGTs, New-DMs and Known-DMs subjects.

We obtained and analyzed 4,111 eubacterial OTUs among the three study groups. Analysis of alpha diversity indices revealed that overall diversity in New-DMs was noticeably reduced and both expected (Chao1, *p* = 0.095) and observed (Observed Species, *p* = 0.047) species diversity indices were lowered in New-DMs and Known-DMs subjects (Figure 1A). Out of eight bacterial phyla detected, Proteobacteria (*p* = 0.026) were significantly lowered, Firmicutes (*p* = 0.010) were significantly higher while Bacteroidetes (*p* = 0.098) showed decreased trend in abundance in New- and Known-DMs subjects (Figure 1B). Kruskal-Wallis test (without *post-hoc* analysis) revealed the presence of 83 significantly different OTUs (*p* < 0.01) of which *Prevotella copri, Faecalibacterium prausnitzii* and Lachnospiraceae OTUs were enriched in NGTs whereas *Lactobacillus ruminis* OTUs were found enriched in Known-DMs (Figure 2A). Moreover, 2 OTUs belonging to genus streptococcus were abundant in New-DMs. Interestingly, the OTUs assigned to *P. copri* and Lachnospiraceae were found to be negatively correlated with fasting glucose (Supplementary Table 2). Using UniFrac distance based PCoA biplots, we demonstrate substantial segregation of the subjects into three groups based on the presence/absence (Unweighted UniFrac, Figure 2B) and abundance of specific bacterial taxa (Weighted UniFrac, Figure 2C).We thus suggest that the presence of discrete clusters of samples in PCoA biplot is an indication of unique bacterial community structure in the three study groups. We further observed that OTUs belonging to order Bacteroidales, family Lachnospiraceae and phylum Bacteroidetes and genus *Prevotella* were determinative taxa for segregation of NGTs from New-DMs and Known-DMs subjects on PCoA biplots. It was noted that *Lactobacillus* was the crucial contributor for segregation of Known-DMs from rest of the samples and thus confirms the findings of Kruskal-Wallis test (performed above) demonstrating enrichment of *L. ruminis* in these subjects. After the Random Forest analysis, top 20 OTUs were considered as highly discriminative among the three groups (Figure 3A). Considering the fact that multiple hypothesis testing was not applied during the Kruskal-Wallis test, we speculate that some of the differences may be overstated. Therefore, we performed qPCR-based absolute quantification to support our major findings of decreased total bacterial count and phylum Bacteroidetes; and increased abundance of genus *Lactobacillus* in New- and Known-DMs subjects (Figure 3B).

### Disease state has profound effect on composition of enterotypes

**Figure 4:**
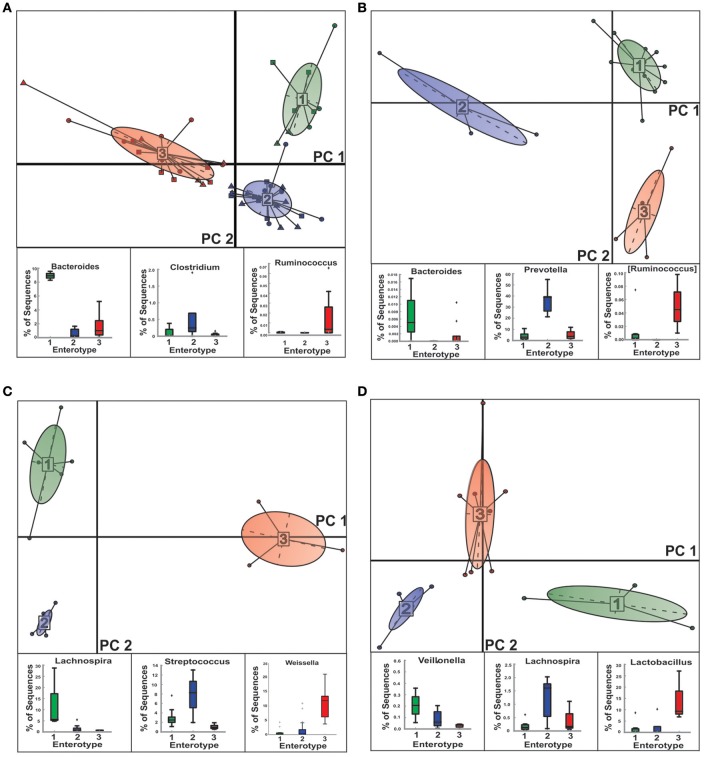
**Clustering of NGTs, New-DMs and Known-DMs subjects into enterotypes (E). (A)** Clustering of all 49 subjects into enterotypes, NGTs (squares), New-DMs (circles), and Known-DMs (Triangles) subjects. **(B)** Clustering of NGTs subjects only. **(C)** Clustering of New-DMs only and **(D)** Clustering of Known-DMs only. Upper panel of each part is showing projection of first two principal components of between-class analysis and lower panel shows the driver genera in corresponding enterotypes (E1, green; E2, blue; and E3, red).

We were able to stratify the gut microbial communities of NGTs, New-DMs as well as Known-DMs subjects into three distinct enterotypes (E) (Figure 4 and Supplementary Figure 1). As observed earlier by Arumugam et al., healthy (NGTs) subjects (Figure 4B) grouped into three enterotypes (E1-Bacteroidetes, E2-*Prevotella*, and E3-Ruminococcus). However, notable compositional changes were observed in enterotypes of both New-DMs (Figure 4C) and Known-DMs (Figure 4D) compared to enterotypes of NGTs subjects. Based on the abundance of the different genera we found that all three enterotypes in these subjects were found to driven by members of Firmicutes (New-DMs: E1-*Lachnospira*, E2-*Streptococcus*, and E3-*Weissella* & Known-DMs: E1-*Veillonella*, E2-*Lachnospira* and E3- *Lactobacillus*). Notably, the E2 (five subjects) in New-DMs and E3 (eight subjects) in Known-DMs were dominated by taxa that were being enriched in these subjects.

### Archaeal, eukaryotic, and fungal dysbiosis

**Figure 5:**
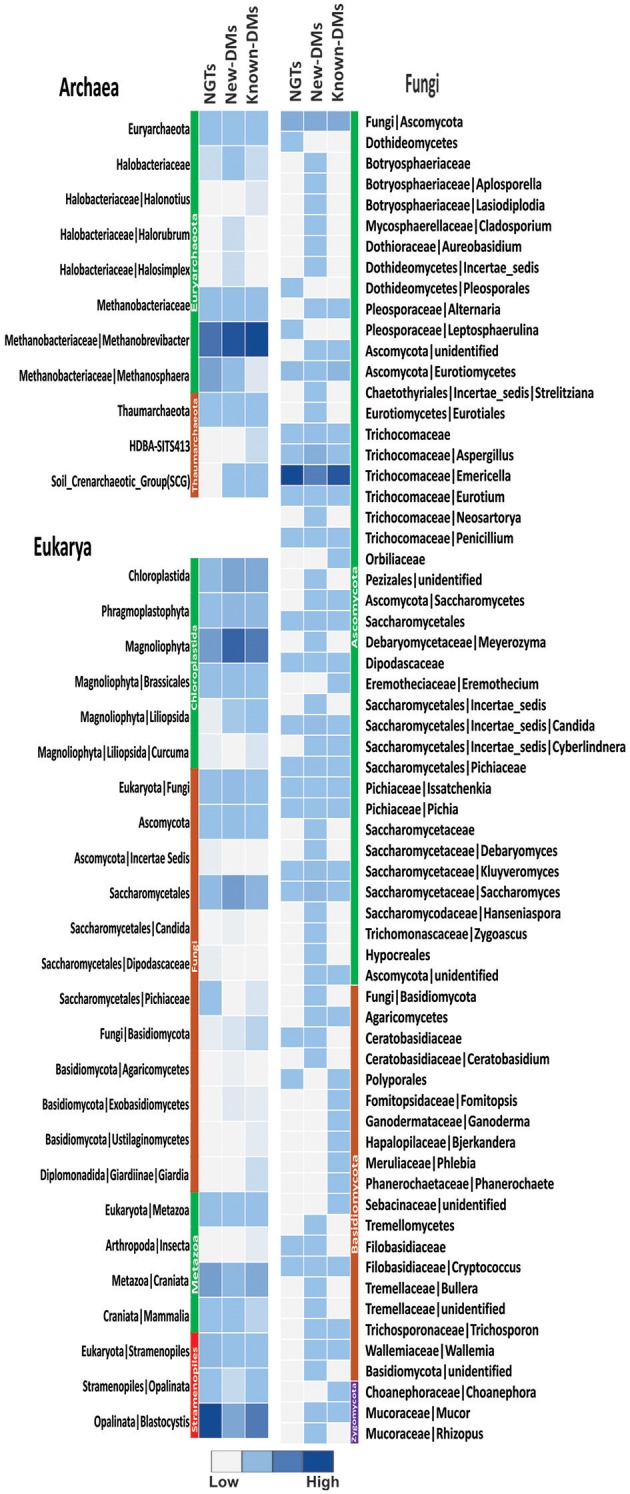
**Assessment of archaea, eukarya, and fungi**. Heatmap showing abundance of different members of archaeal, eukaryal and fungal components of NGTs, New-DMs and Known-DMs subjects.

We generated 109,561 good quality archaeal 16S rRNA amplicon reads from three pools of samples (NGTs, New-DMs, and Known-DMs); which clustered into 65 OTUs belonging to Euryarchaeota and Thaumarchaeota phyla. The former being the most dominated phylum occupying more than 99% reads of all three groups. We noticed the gradual increase in *Methanobrevibacter* (which was also the most abundant taxa in all groups) and associated decrease in *Methanosphaera* abundance from NGTs to New-DMs to Known-DMs subjects. From the three pools of Eukaryotic sequence data, we obtained 41,959 good quality sequences that clustered into 383 OTUs and could be assigned to four phyla: Chloroplastida, Metazoa, Stramenopiles, and Metamonada. Members of Stramenopile especially members of genus *Blastocystis* were found abundant in all groups. Fungi, particularly members belonging to Saccharomycetales were abundant in New-DMs compare to NGTs and Known-DMs. For fungal ITS data, we could obtain 106,185 reads that clustered into 871 OTUs belonging to phyla Ascomycota being most dominant followed by Basidiomycota and Zygomycota to be least dominant. From the Ascomycota group; *Aspergillus* and *Emericella*, the two alternative forms of the same fungus predominated most of the sequences (Figure 5).

### Altered microbial composition is associated with clinical parameters

**Figure 6:**
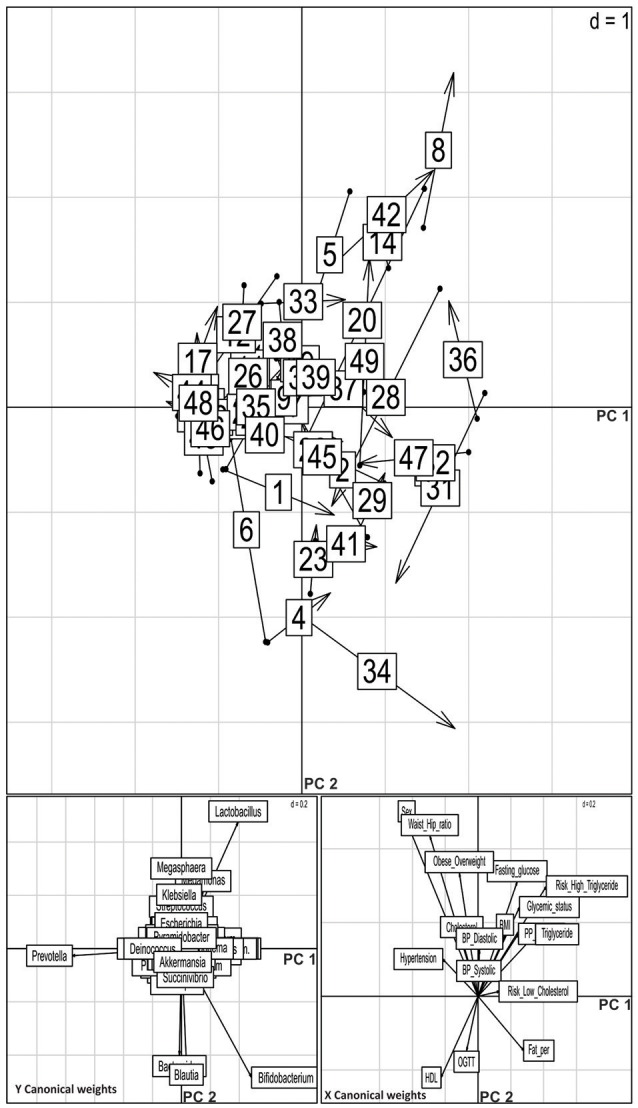
**Co-inertia analysis of relationship of genus level abundance and clinical parameters**. Upper panel shows positions of the site on the co-inertia axes using genus (origin of the arrow) and clinical parameter (arrowheads) co-inertia weights. The shorter the arrow, the better the concordance between the two projections. The numbers indicate the samples: NGTs, 1–19; New-DMs, 20–33; Known-DMs, 34–49. Lower pair of plot shows contribution of the two groups of variable to the canonical space; vectors pointing in the same direction are correlated.

To analyse the effect of different biochemical and anthropometric measurements on sampled microbiota among the three groups, we used PERMANOVA and Co-inertia analysis. After applying PERMANOVA test, we discovered that HDL (*p* = 0.03), triglyceride (*p* = 0.05), and waist-hip ratio (*p* = 0.02) to be associated with OTU diversity across all samples (Supplementary Table 3). In the case of Known-DMs, we found HDL (*p* = 0.01), and in the case of New-DMs, oral glucose tolerance test (*p* = 0.05) to have an influence on distinct OTU diversity. Further, the covariance between genus abundance and clinical and anthropometric parameters were examined using co-inertia analysis (1,000 permutations) of these datasets, resulting in a relationship with RV coefficient = 0.219, *P* = 0.196 between the datasets (Figure 6). Similar and subsequent analysis were not performed on simulated datasets of Archaeal, Eukaryotic and Fungal datasets.

### Eubacterial interaction network

**Figure 7:**
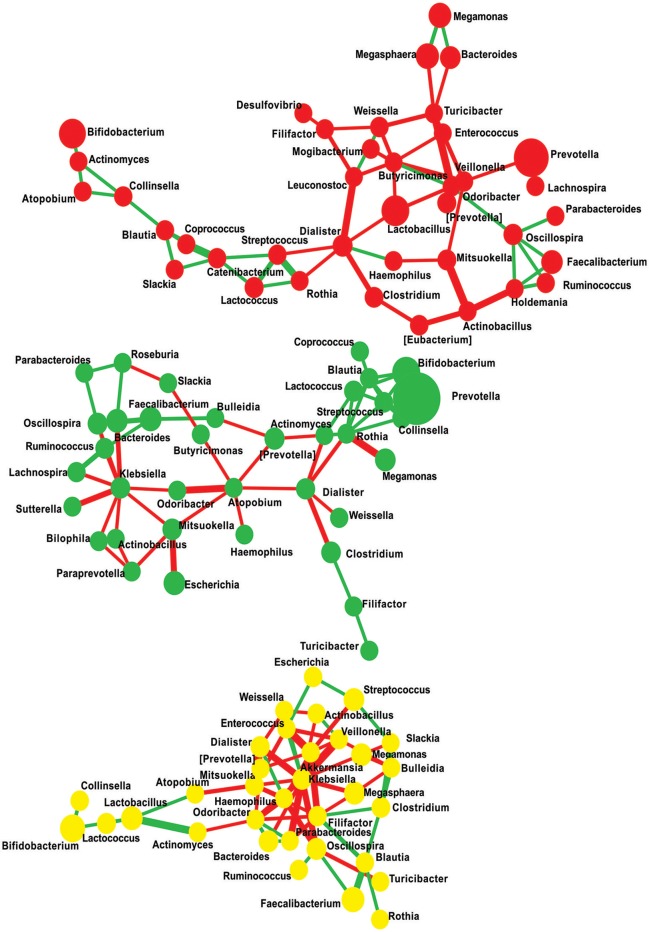
**Significant co-occurrence and co-exclusion relationships at genus level**. Each node represents a bacterial genus; size of the node is proportional to the abundance of the genus and colored according to diabetes status (Red: Known-DMs, Yellow: New-DMs, and Green: NGTs). Each edge represents co-occurrence/co-exclusion relationships, edge width is proportional to the significance of supporting evidence, and color indicates sign of the association (red: negative, green: positive).

Microbiome network containing a total of 108 nodes connected with 174 edges together representing 46% co-occurrence and 54% of mutual exclusion interactions were obtained. Further, to measure the scale-freeness of the network, we used fitted power law and obtained correlation of 0.6 with the R-square value of 0.723 (Supplementary Figure 2). This network reveals that the patterns observed were disease state specific, i.e., majority of the edges were found clustering within one study group providing a clue that individuals in each group have distinctly interacting microbiome composition (Figure 7 and Supplementary Table 4). We then filtered the network to retain nodes positively interacting with each other, assuming that microbes represented by these nodes will stay together in a given community. In the filtered network of positively interacting genera, we noticed that a cluster of *Lachnospira, Ruminococcus, Faecalibacterium, Roseburia, Oscillospira, Parabacteroides*, and *Bulleidia* decomposed from NGTs to New-DMs then to Known-DMs (Supplementary Figures 3–5). We also noted negative interactions of *Lactobacillus* in Known-DMs.

### Deficient metabolic activities in new-DMs as revealed by imputed metagenome

Having identified the compositional changes in microbiota with respect to diabetes state, we tested whether these changes are accompanied with selectively fostering or lacking particular functional capabilities of gut microbiota. Similarities and differences in metabolic capabilities in gut microbiota were evaluated by making the pair-wise comparison between the diabetes statuses using two-sided Welch's *t*-test. Compared to NGTs, the metagenome of New-DMs was found augmented with glycerolipid metabolism, fructose and mannose metabolism, pentose phosphate pathway, galactose metabolism, glycolysis/gluconeogenesis and arginine and proline metabolism. Concurrently, these subjects were found to be deficient in many important metabolic activities such as carbohydrate metabolism (including carbohydrate digestion and absorption, TCA cycle, oxidative phosphorylation, glycan biosynthesis and metabolism, glycosyltransferases), amino acid metabolism (including metabolism of glycine, serine, threonine, histidine), vitamin B metabolism (including folate, biotin, pyridoxine metabolism), glutathione metabolism and other functions (Supplementary Figure 6). Compared to Known-DMs, New-DMs were deficient of carbohydrate digestion and absorption, glycosyltransferases and glutathione metabolism (Supplementary Figure 7). Conversely, they were enriched with functions unrelated to carbohydrate or amino acid or lipid metabolism compared to NGTs (Supplementary Figure 8).

## Discussion

The present study is first to report perturbation in the gut microbiota of Indian diabetic subjects across the three domains of life. Considering the unique characteristics of Indian diabetic subjects, understanding their gut microbiota will be important to understand the possible role of gut microbiota in affecting these characteristics. Members of eubacteria such as *P. copri*, Lachnospiraceae and Ruminococcaceae families were found significantly abundant in NGTs subjects. Known-DMs subjects exhibited increased abundance of Firmicutes and OTUs belonging to genus *Lactobacillus*. These organisms were seen to have an effect on the segregation of samples in both unweighted and weighted UniFrac based PCoA biplots. Fungi prevailed in New-DMs; in particular, genera *Aspergillus, Candida*, and *Saccharomyces* were found enriched in these subjects. We also observed the progressive decline in butyrate-producing bacteria from NGTs to Known-DMs subjects. These variations in gut microbiota were associated with diabetes risk factors such as fasting glucose, high triglycerides, low HDL and fasting insulin. Additionally, synergistic or antagonistic interactions occurring in gut microbiota were found specific to the stage of glucose intolerance. Using PICRUSt, we predicted that the gut microbiome of New-DMs subjects was metabolically disturbed and was lacking in many necessary functions.

Increased Firmicutes and proportionate decrease in Bacteroidetes is linked with more energy harvesting and storage in ob/ob animals (Turnbaugh et al., 2006). Analogous to animal studies, human obesity is also found to be linked with higher Firmicutes to Bacteroidetes ratio (Ley et al., 2006). Our finding of increased abundance of Firmicutes in known-DMs is in agreement with previous reports (Karlsson et al., 2013) but not with findings of Larsen and co-workers, who reported a decrease in the proportion of Firmicutes (Larsen et al., 2010). Association of Firmicutes with obesity and diabetes could operate through insulin resistance which is a common attribute of both the conditions (Pandolfi et al., 1994).

Analysis of differentially abundant OTUs revealed that NGTs were highly enriched with Prevotellaceae, Lachnospiraceae, and Ruminococcaceae families. Members belonging to Prevotellaceae such as genus *Prevotella* contribute significantly to inter-individual variation in gut microbiota (Arumugam et al., 2011) and increased proportions of *Prevotella* are associated with the diet rich in plant-derived complex carbohydrates and fibers such as the diet in Indians (De Filippo et al., 2010). Additionally, a study in which subjects were kept of dietary interventions (barley kernel-based bread, which is considered as a rich source of fibers), showed that there was a significant increase in *P. copri* and that it was found to be associated with improvement in glucose metabolism in these subjects (Kovatcheva-Datchary et al., 2015). Strikingly, several studies on type 1 diabetes, a pathophysiologically different disorder related to persistent hyperglycemia, are also reporting reduced levels of *Prevotella* in newly diagnosed as well as longstanding type 1 diabetic subjects (Mejía-León et al., 2014; Alkanani et al., 2015; Mejía-León and Barca, 2015). At this moment we could speculate that this could just be a coincidence or indeed it is linked with hyperglycemia *per se* which is a common attribute of type 1 and type 2 diabetes. Members of families Lachnospiraceae and Ruminococcaceae are known producers of short-chain fatty acids (SCFAs) such as acetate and butyrate. These SCFAs are known to confer many health benefits; individuals lacking bacterial families producing SCFAs suffer from many diseases (Morgan et al., 2012). Interestingly, we observed decreasing trends in the richness of these bacterial families with progressive deterioration of glucose tolerance (from NGTs to New-DMs to Known-DMs subjects). Presence of these families in the gut may be essential to foster a “healthy state,” and their depletion might have a role in diabetes development (Remely et al., 2014). Thus, we hypothesize that the decreased abundance of *P. copri* and concomitant loss of short chain fatty acids producers in New- and Known-DMs subjects could be linked with glucose intolerance in these subjects as these organisms were found to be negatively correlated with fasting glucose in our analyses.

We also found that Known-DMs were enriched with genus *Lactobacillus* consistent with previous studies on diabetic subjects in different populations of the world (Larsen et al., 2010; Lê et al., 2012). Karlsson and co-workers have also demonstrated enrichment of lactobacilli-derived metagenomic clusters (MGCs) in type 2 diabetic patients that they found positively correlating with fasting glucose and HbA_1c_. Another large-scale study dealing with the characterization of over 170 *Lactobacillus* species from oral cavity showed a higher prevalence of lactobacilli in diabetic subjects (Teanpaisan et al., 2009) and this increase in *Lactobacillus* species has been linked with increased salivary glucose in children with diabetes (Karjalainen et al., 1996). *Lactobacillus ruminis*, which we found significantly abundant in Known-DMs subjects, is a member of indigenous gut microflora (O' Donnell et al., 2015). It has approximately 16 carbohydrate utilization pathways including those for utilization of glucose, fructose, mannose, galactose, starch, and sucrose (Forde et al., 2011). Thus, as reported earlier, the catabolic flexibility of this organism toward varied dietary carbohydrates is evident (O'Donnell et al., 2011). Above facts taken together, indicate that enrichment of the lactobacilli in gastrointestinal tract of diabetic subjects could be a consequence of higher than usual concentration of glucose, which needs to be confirmed.

Besides this, we also show the gradation of NGTs, New-DMs and Known-DMs samples on UniFrac biplots. These UniFrac biplots were plotted using phylogenetic distance which is calculated utilizing unique branch-lengths i.e., only those branches that lead to descendants from one or the other sample but not both samples in a phylogenetic tree were considered (Lozupone et al., 2011). Hence, we believe that segregation of the samples is robust and could be because of the above mentioned compositional differences in bacterial communities in these subjects. We next attempted to group study participants into distinct clusters based on the presence of unique and dominant gut microbial communities called “enterotypes” (Arumugam et al., 2011). Currently, the concept of enterotype is generating a lot of debate; different groups have different opinions about the presence or absence of such discrete cluster in human gut microbiome (Knights et al., 2014; Moeller et al., 2015). Although, it has been shown earlier that during identification of enterotypes, various factors influence clustering of subjects into distinct enterotypes (Koren et al., 2013); we feel that it is beyond the reach of this article to deal with theories of formation of enterotypes and associated factors affecting their formation, hence, we performed this analysis as originally proposed (Arumugam et al., 2011). We find substantial changes in major contributors of enterotype in New- and Known-DMs subjects compared to NGTs subjects. Especially, we observed E2 in New-DMs and E3 in Know-DMs subjects to be driven by *Streptococcus* and *Lactobacillus* respectively. These findings are important because clustering of subjects based on the presence of unique and predominated taxa could help us in identifying disease-related biomarkers, thus it can find its implications in microbiome-based diagnostics (Knights et al., 2014).

We next looked into archaeal diversity in the three sample groups; *Methanobrevibacter* and *Methanosphaera* were the most prevalent genera. *Methanobrevibacter smithii* (*M. smithii*) and *Methanosphaera stadtmanae* are well adapted to the human gut environment, interestingly, the latter has acquired most of these adaptations through inter-domain lateral gene transfer (Samuel et al., 2007; Lurie-Weinberger et al., 2012). As perceived by us and reported in a previous study (Turnbaugh et al., 2006), *Methanobrevibacter smithii* has been represented in large proportion along with increased Firmicutes; it was involved in increased energy harvest through polysaccharide degradation. Further, the same study noted that this attribute was transmissible such that microbiota transplantation from obese donor to lean germ-free mice lead to the gain in body fat. Additionally, *Methanobrevibacter smithii* directs polysaccharide utilization by gut inhabitants, leading to the formation of large pools of SCFAs which is later used by *M. smithii* for methanogenesis in the gut with a consequent increase in host adiposity (Samuel and Gordon, 2006). Thus, *Methanobrevibacter smithii* can be a therapeutic target to avoid obesity and associated complications such as diabetes (Samuel et al., 2007).

Based on the work we carried out and several other similar studies, gut eukaryotes and fungi appear to be important components of the human gut. Such studies are crucial in the light of the involvement of these organisms in human diseases both inside and outside of gastrointestinal tract (Cui et al., 2013). Morphological and molecular phylogenetic-based classification of eukaryotes show that all eukaryotes originate from one of the six super-groups and that most of them are microscopic in nature (Adl et al., 2005). Although for decades human-associated eukaryotes are considered harmful to their host, recent examination of eukaryotic communities in the gut are amending our understanding of this generally neglected component (Hamad et al., 2012; Pandey et al., 2012; Parfrey et al., 2014). Studies such as these and our findings suggest that *Blastocystis* and fungi such as Ascomycota and Basidiomycota are predominant in the human gut. Fungi such as *Candida albicans, Aspergillus fumigatus*, and *Saccharomyces* are opportunistic pathogens known to be exaggerated in immune-compromised people (Li E. et al., 2014; Gouba and Drancourt, 2015). Fungal species mentioned above have also been associated with various diseases in type 1 (Soyucen et al., 2014) and type 2 diabetic subjects (Aly et al., 1991; Nowakowska et al., 2004) and are probably because of the high blood glucose level in these subjects. Thus, marked enrichment of fungi belonging to these and other genera in New-DMs subjects are likely due to the poor glycemic control in these subjects.

We investigated associations between clinical parameters and OTU richness using permutational multivariate analysis of variance (PERMANOVA). PERMANOVA is considered a powerful technique in detecting changes in community structure in response to environmental parameters (Anderson and Walsh, 2013). We observed that HDL, triglyceride and waist-hip ratio as largest contributors to the observed variation in OTU richness. Such correlations between risk factors for diabetes and variation in microbes in the gut have been previously reported (Zhang et al., 2013) and are also reflected in our dataset. Thus, it could be relevant in the microbiome-phenotype associations, since, low HDL and high triglycerides are typical features of dyslipidaemia found in T2D and known risk factors for cardiovascular disease (Mooradian, 2009).

We used network analysis to capture specific ecological interactions among the eubacterial consortium in relation to diabetes status. Such interaction networks can predict the outcome of community alterations (Faust et al., 2012) and be helpful in designing intervention studies aimed at altering complex microbial communities to restore the healthy state. In essence, we are not demonstrating complete coverage of all microbial interactions in the gut; but analyzing the interactions among microbes in the gut will help us understand how these communities develop or evolve in response to altered physiological and/or metabolic state such as diabetes. We thus highlight two characteristic features of this network: (1) the nature of the interactions observed were diabetes state specific and (2) the disintegration of the microbial cluster of genera: *Lachnospira, Ruminococcus, Faecalibacterium, Roseburia, Oscillospira, Parabacteroides, Bulleidia* from NGTs to New-DMs to Known-DMs. Almost all these genera include known beneficial species having the ability to produce SCFAs as mentioned earlier. Importantly, metagenomic linkage clusters (MLGs) belonging to these butyrate-producing genera were found enriched in non-diabetic controls in diabetes associated metagenomic study (Qin et al., 2012).

Finally, with the bioinformatics tool PICRUSt (Langille et al., 2013) which predicts functional composition using marker gene data, we had an opportunity to look into imputed metagenome-based discrete functional alteration in the eubacterial component of our study subjects. We observed that New-DMs were severely depleted with metabolic functions involved in carbohydrate metabolism, amino acid metabolism, various cofactor synthesis and oxidative stress management. Although PICRUSt can accurately predict metagenomic functions, it is limited to those sequences that can be accurately mapped to existing Greengenes database and does not consider sequences from novel microbial lineages (Langille et al., 2013). Thus, our explanation on imputed metagenome is limited and interpreted cautiously.

One of the strengths of our study is the comparison of gut microbiota of different grades of glucose intolerant subjects from a cohort which is has been followed for the past 20 years, this allowed a confident separation between newly diagnosed and known diabetic subjects. The participants are from the similar socioeconomic background and have a predominantly vegetarian diet. The age and gender distribution in the three groups were similar. One of the limitations of this study is that we were unable to describe sequential events in gut microbiota from healthy to diabetic state due to the cross-sectional design of this study. Another limitation of the study is the relatively small number of participants from one part of the country. Given the diversity in lifestyles, dietary habits, and social-economic status in the country, this study underscores a need for nationwide longitudinal studies. Our study is subject to inherent biases introduced by the use of high-throughput 16S rRNA gene amplicon sequencing. These include the region of 16S rRNA gene sequenced, set of primers used for gene amplification and use of sequence database for taxonomic assignments of the amplicon reads.

In conclusion, our results add to the growing literature suggesting an association between gut microbiota and diabetes. Broad similarities between our results and literature reports suggest that our measurements are reliable and support consistent association across populations. Additionally, we have broadened the boundaries of diabetes associated gut microbiota by providing the consolidated description on eubacterial, archaeal, and eukaryotic dysbiosis in these subjects. Given the peculiarities of diabetes in Indians, these results suggest an important avenue be further explored for causality and possible interventions to prevent or modify the course of diabetes and related disorders. We anticipate the need for subsequent studies describing differences in gut microbial communities of diabetic patients from different populations and identification of relevant population specific biomarkers.

## Author contributions

YS, SG, and CY contributed to conception, design, and coordination of the study and to the critical revisions of the manuscript for important intellectual content. SJ and CY were involved in subject recruitment and sample collection. SB acquired and processed the fecal samples for 16S rRNA amplicon sequencing. SB performed detailed bioinformatics analysis. SB and MS performed archaeal, eukaryotic and fungal amplicon sequencing. SB prepared the first draft of the manuscript and contributed to the critical revisions of the manuscript for important intellectual content. SB and SJ undertook statistical analysis and interpretation of results. SJ contributed to the critical revisions of the manuscript for important intellectual content. All authors gave final approval of the version to be published.

### Conflict of interest statement

The authors declare that the research was conducted in the absence of any commercial or financial relationships that could be construed as a potential conflict of interest.

## References

[B1] Adl S. M. Simpson A. G. B. Farmer M. A. Andersen R. A. Anderson O. R. Barta J. R.. (2005). The new higher level classification of eukaryotes with emphasis on the taxonomy of protists. J. Eukaryot. Microbiol. 52, 399–451. 10.1111/j.1550-7408.2005.00053.x 16248873

[B2] Alberti K. G. Zimmet P. Z. (1998). Definition, diagnosis and classification of diabetes mellitus and its complications, part 1: diagnosis and classification of diabetes mellitus provisional report of a WHO consultation. Diabet. Med. 15, 539–553. 10.1002/(SICI)1096-9136(199807)15:7<539::AID-DIA668>3.0.CO;2-S 9686693

[B3] Alkanani A. K. Hara N. Gottlieb P. A. Ir D. Robertson C. E. Wagner B. D. (2015). Alterations in intestinal microbiota correlate with susceptibility to type 1 diabetes. Diabetes 64, 3510–3520. 10.2337/db14-1847 26068542 PMC4587635

[B4] Aly F. Z. Blackwell C. C. MacKenzie D. A. Weir D. M. Elton R. A. Cumming C. G.. (1991). Chronic atrophic oral candidiasis among patients with diabetes mellitus–role of secretor status. Epidemiol. Infect. 106, 355–363. 10.1017/S0950268800048500 2019303 PMC2272001

[B5] Anderson M. J. Walsh D. C. I. (2013). PERMANOVA, ANOSIM, and the Mantel test in the face of heterogeneous dispersions- What null hypothesis are you testing? Ecol. Monogr. 83, 557–574. 10.1890/12-2010.1

[B6] Anjana R. M. Pradeepa R. Deepa M. Datta M. Sudha V. Unnikrishnan R.. (2011). Prevalence of diabetes and prediabetes (impaired fasting glucose and/or impaired glucose tolerance) in urban and rural India: phase I results of the Indian Council of Medical Research-INdia DIABetes (ICMR-INDIAB) study. Diabetologia 54, 3022–3027. 10.1007/s00125-011-2291-5 21959957

[B7] Arumugam M. Raes J. Pelletier E. Le Paslier D. Yamada T. Mende D. R.. (2011). Enterotypes of the human gut microbiome. Nature 473, 174–180. 10.1038/nature09944 21508958 PMC3728647

[B8] Arumugam M. Raes J. Pelletier E. Le Paslier D. Yamada T. Mende D. R. (2014). Addendum: enterotypes of the human gut microbiome. Nature 506, 516–516. 10.1038/nature13075 21508958 PMC3728647

[B9] Bartram A. K. Lynch M. D. J. Stearns J. C. Moreno-Hagelsieb G. Neufeld J. D. (2011). Generation of multimillion-sequence 16S rRNA gene libraries from complex microbial communities by assembling paired-end Illumina reads. Appl. Environ. Microbiol. 77, 3846–3852. 10.1128/AEM.02772-10 21460107 PMC3127616

[B10] Bhute S. Pande P. Shetty S. A. Shelar R. Mane S. Kumbhare S. V.. (2016). Molecular characterization and meta-analysis of gut microbial communities illustrate enrichment of prevotella and megasphaera in Indian subjects. Front. Microbiol. 7:660. 10.3389/fmicb.2016.00660 27242691 PMC4860526

[B11] Caporaso J. G. Kuczynski J. Stombaugh J. Bittinger K. Bushman F. D. Costello E. K. (2010). QIIME allows analysis of high- throughput community sequencing data Intensity normalization improves color calling in SOLiD sequencing. Nat. Methods 7, 335–336. 10.1038/nmeth.f.303 20383131 PMC3156573

[B12] Cénit M. C. Matzaraki V. Tigchelaar E. F. Zhernakova A. (2014). Rapidly expanding knowledge on the role of the gut microbiome in health and disease. Biochim. Biophys. Acta 1842, 1981–1992. 10.1016/j.bbadis.2014.05.023 24882755

[B13] Chao A. (1984). Nonparametric-estimation of the number of classes in a population.pdf. Scan. J. Statisit. 11, 265–270.

[B14] Clemente J. C. Ursell L. K. Parfrey L. W. Knight R. (2012). The impact of the gut microbiota on human health: an integrative view. Cell 148, 1258–1270. 10.1016/j.cell.2012.01.035 22424233 PMC5050011

[B15] Cui L. Morris A. Ghedin E. (2013). The human mycobiome in health and disease. Genome Med. 5, 63. 10.1186/gm467 23899327 PMC3978422

[B16] De Filippo C. Cavalieri D. Di Paola M. Ramazzotti M. Poullet J. B. Massart S.. (2010). Impact of diet in shaping gut microbiota revealed by a comparative study in children from Europe and rural Africa. Proc. Natl. Acad. Sci. U.S.A. 107, 14691–14696. 10.1073/pnas.1005963107 20679230 PMC2930426

[B17] Dollive S. Peterfreund G. L. Sherrill-Mix S. Bittinger K. Sinha R. Hoffmann C.. (2012). A tool kit for quantifying eukaryotic rRNA gene sequences from human microbiome samples. Genome Biol. 13, 1–13. 10.1186/gb-2012-13-7-r60 22759449 PMC4053730

[B18] Dray S. Chessel D. Thioulouse J. (2003). Co-inertia analysis and the linking of ecological data tables. Ecology 84, 3078–3089. 10.1890/03-0178

[B19] Faust K. Sathirapongsasuti J. F. Izard J. Segata N. Gevers D. Raes J.. (2012). Microbial co-occurrence relationships in the human microbiome. PLoS Comput. Biol. 8:e1002606. 10.1371/journal.pcbi.1002606 22807668 PMC3395616

[B20] Forde B. M. Neville B. A. O'Donnell M. M. Riboulet-Bisson E. Claesson M. J. Coghlan A.. (2011). Genome sequences and comparative genomics of two *Lactobacillus ruminis* strains from the bovine and human intestinal tracts. Microb. Cell Fact. 10(Suppl. 1):S13. 10.1186/1475-2859-10-S1-S13 21995554 PMC3231920

[B21] Frank D. N. St. Amand A. L. Feldman R. A. Boedeker E. C. Harpaz N. Pace N. R. (2007). Molecular-phylogenetic characterization of microbial community imbalances in human inflammatory bowel diseases. Proc. Natl. Acad. Sci. U.S.A. 104, 13780–13785. 10.1073/pnas.0706625104 17699621 PMC1959459

[B22] Gaci N. Borrel G. Tottey W. O'Toole P. W. Brugère J.-F. (2014). Archaea and the human gut: new beginning of an old story. World J. Gastroenterol. 20, 16062–16078. 10.3748/wjg.v20.i43.16062 25473158 PMC4239492

[B23] Gouba N. Drancourt M. (2015). Digestive tract mycobiota: a source of infection. Méd. Mal. Infect. 45, 9–16. 10.1016/j.medmal.2015.01.007 25684583

[B24] Gouba N. Raoult D. Drancourt M. (2014). Gut microeukaryotes during anorexia nervosa: a case report. BMC Res. Notes 7:33. 10.1186/1756-0500-7-33 24418238 PMC3895777

[B25] Grattepanche J.-D. Santoferrara L. F. McManus G. B. Katz L. A. (2014). Diversity of diversity: conceptual and methodological differences in biodiversity estimates of eukaryotic microbes as compared to bacteria. Trends Microbiol. 22, 432–437. 10.1016/j.tim.2014.04.006 24814699

[B26] Hamad I. Sokhna C. Raoult D. Bittar F. (2012). Molecular detection of eukaryotes in a single human stool sample from senegal. PLoS ONE 7:e40888. 10.1371/journal.pone.0040888 22808282 PMC3396631

[B27] International Diabetes Federation (2015). Idf Diabetes Atlas. Diabetes Atlas. Available online at: http://www.diabetesatlas.org/

[B28] Karjalainen K. M. Knuuttila M. L. Käär M. L. (1996). Salivary factors in children and adolescents with insulin-dependent diabetes mellitus. Pediatr. Dent. 18, 306–311. 8857659

[B29] Karlsson F. H. Tremaroli V. Nookaew I. Bergström G. Behre C. J. Fagerberg B.. (2013). Gut metagenome in European women with normal, impaired and diabetic glucose control. Nature 498, 99–103. 10.1038/nature12198 23719380

[B30] Knights D. Ward T. L. McKinlay C. E. Miller H. Gonzalez A. McDonald D.. (2014). Rethinking “enterotypes”. Cell Host Microbe 16, 433–437. 10.1016/j.chom.2014.09.013 25299329 PMC5558460

[B31] Kõljalg U. Nilsson R. H. Abarenkov K. Tedersoo L. Taylor A. F. S. Bahram M.. (2013). Towards a unified paradigm for sequence-based identification of fungi. Mol. Ecol. 22, 5271–5277. 10.1111/mec.12481 24112409

[B32] Koren O. Knights D. Gonzalez A. Waldron L. Segata N. Knight R.. (2013). A guide to enterotypes across the human body: meta-analysis of microbial community structures in human microbiome datasets. PLoS Comput. Biol. 9:e1002863. 10.1371/journal.pcbi.1002863 23326225 PMC3542080

[B33] Kovatcheva-Datchary P. Nilsson A. Akrami R. Lee Y. S. De Vadder F. Arora T.. (2015). Dietary fiber-induced improvement in glucose metabolism is associated with increased abundance of Prevotella. Cell Metab. 22, 971–982. 10.1016/j.cmet.2015.10.001 26552345

[B34] Langille M. G. I. Zaneveld J. Caporaso J. G. McDonald D. Knights D. Reyes J. A.. (2013). Predictive functional profiling of microbial communities using 16S rRNA marker gene sequences. Nat. Biotechnol. 31, 814–821. 10.1038/nbt.2676 23975157 PMC3819121

[B35] Larsen N. Vogensen F. K. van den Berg F. W. J. Nielsen D. S. Andreasen A. S. Pedersen B. K.. (2010). Gut microbiota in human adults with type 2 diabetes differs from non-diabetic adults. PLoS ONE 5:e9085. 10.1371/journal.pone.0009085 20140211 PMC2816710

[B36] Lê K.-A. Li Y. Xu X. Yang W. Liu T. Zhao X.. (2012). Alterations in fecal Lactobacillus and Bifidobacterium species in type 2 diabetic patients in Southern China population. Front. Physiol. 3:496. 10.3389/fphys.2012.00496 23386831 PMC3560362

[B37] Lepp P. W. Brinig M. M. Ouverney C. C. Palm K. Armitage G. C. Relman D. A. (2004). Methanogenic Archaea and human periodontal disease. Proc. Natl. Acad. Sci. U.S.A. 101, 6176–6181. 10.1073/pnas.0308766101 15067114 PMC395942

[B38] Ley R. E. Hamady M. Lozupone C. Turnbaugh P. J. Ramey R. R. Bircher J. S.. (2008). Evolution of mammals and their gut microbes. Science 320, 1647–1651. 10.1126/science.1155725 18497261 PMC2649005

[B39] Ley R. Turnbaugh P. Klein S. Gordon J. (2006). Microbial ecology: human gut microbes associated with obesity. Nature 444, 1022–1023. 10.1038/4441022a 17183309

[B40] Li E. Hussein H. Todiwala A. Kirby R. (2014). Primary gut aspergillosis in a patient with acute myeloid leukaemia: the importance of early suspicion and definitive treatment. BMJ Case Rep. 2014:bcr2013202316. 10.1136/bcr-2013-202316 24642177 PMC3962853

[B41] Li Q. Wang C. Tang C. He Q. Li N. Li J. (2014). Dysbiosis of gut fungal microbiota is associated with mucosal inflammation in crohn's disease. J. Clin. Gastroenterol. 48, 513–523. 10.1097/MCG.0000000000000035 24275714 PMC4059552

[B42] Lozupone C. Lladser M. E. Knights D. Stombaugh J. Knight R. (2011). UniFrac: an effective distance metric for microbial community comparison. ISME J. 5, 169–172. 10.1038/ismej.2010.133 20827291 PMC3105689

[B43] Luan C. Xie L. Yang X. Miao H. Lv N. Zhang R.. (2015). Dysbiosis of fungal microbiota in the intestinal mucosa of patients with colorectal adenomas. Sci. Rep. 5:7980. 10.1038/srep07980 25613490 PMC4648387

[B44] Lucke K. Miehlke S. Jacobs E. Schuppler M. (2006). Prevalence of *Bacteroides* and *Prevotella* spp. in ulcerative colitis. J. Med. Microbiol. 55, 617–624. 10.1099/jmm.0.46198-0 16585651

[B45] Lurie-Weinberger M. N. Peeri M. Tuller T. Gophna U. (2012). Extensive inter-domain lateral gene transfer in the evolution of the human commensal *Methanosphaera stadtmanae*. Front. Genet. 3:182. 10.3389/fgene.2012.00182 23049536 PMC3445992

[B46] Mejía-León M. E. Barca A. M. (2015). Diet, Microbiota and immune system in type 1 diabetes development and evolution. Nutrients 7, 9171–9184. 10.3390/nu7115461 26561831 PMC4663589

[B47] Mejía-León M. E. Petrosino J. F. Ajami N. J. Domínguez-Bello M. G. de la Barca A. M. C. (2014). Fecal microbiota imbalance in Mexican children with type 1 diabetes. Sci. Rep. 4:3814. 10.1038/srep03814 24448554 PMC3898044

[B48] Million M. Maraninchi M. Henry M. Armougom F. Richet H. Carrieri P.. (2012). Obesity-associated gut microbiota is enriched in Lactobacillus reuteri and depleted in Bifidobacterium animalis and *Methanobrevibacter smithii*. Int. J. Obes. 36, 817–825. 10.1038/ijo.2011.153 21829158 PMC3374072

[B49] Moeller A. H. Peeters M. Ayouba A. Ngole E. M. Esteban A. Hahn B. H.. (2015). Stability of the gorilla microbiome despite simian immunodeficiency virus infection. Mol. Ecol. 24, 690–697. 10.1111/mec.13057 25545295 PMC4302016

[B50] Mooradian A. D. (2009). Dyslipidemia in type 2 diabetes mellitus. Nat. Clin. Pract. Endocrinol. Metab. 5, 150–159. 10.1038/ncpendmet1066 19229235

[B51] Morgan X. C. Tickle T. L. Sokol H. Gevers D. Devaney K. L. Ward D. V.. (2012). Dysfunction of the intestinal microbiome in inflammatory bowel disease and treatment. Genome Biol. 13:R79. 10.1186/gb-2012-13-9-r79 23013615 PMC3506950

[B52] Nowakowska D. Kurnatowska A. Stray-Pedersen B. Wilczyński J. (2004). Species distribution and influence of glycemic control on fungal infections in pregnant women with diabetes. J. Infect. 48, 339–46. 10.1016/j.jinf.2004.01.017 15066336

[B53] O'Donnell M. M. Forde B. M. Neville B. Ross P. R. O'Toole P. W. (2011). Carbohydrate catabolic flexibility in the mammalian intestinal commensal *Lactobacillus ruminis* revealed by fermentation studies aligned to genome annotations. Microb. Cell Fact. 10(Suppl. 1):S12. 10.1186/1475-2859-10-S1-S12 21995520 PMC3231919

[B54] O' Donnell M. M. Harris H. M. B. Lynch D. B. Ross R. P. O'Toole P. W. (2015). *Lactobacillus ruminis* strains cluster according to their mammalian gut source. BMC Microbiol. 15, 80. 10.1186/s12866-015-0403-y 25879663 PMC4393605

[B55] Pandey P. K. Siddharth J. Verma P. Bavdekar A. Patole M. S. Shouche Y. S. (2012). Molecular typing of fecal eukaryotic microbiota of human infants and their respective mothers. J. Biosci. 37, 221–226. 10.1007/s12038-012-9197-3 22581327

[B56] Pandolfi C. Pellegrini L. Sbalzarini G. Mercantini F. (1994). Obesity and insulin resistance. Minerva Med. 85, 167–171. 8028743

[B57] Parfrey L. W. Walters W. A. Lauber C. L. Clemente J. C. Berg-Lyons D. Teiling C.. (2014). Communities of microbial eukaryotes in the mammalian gut within the context of environmental eukaryotic diversity. Front. Microbiol. 5:298. 10.3389/fmicb.2014.00298 24995004 PMC4063188

[B58] Parks D. H. Beiko R. G. (2010). Identifying biologically relevant differences between metagenomic communities. Bioinformatics 26, 715–721. 10.1093/bioinformatics/btq041 20130030

[B59] Patil D. P. Dhotre D. P. Chavan S. G. Sultan A. Jain D. S. Lanjekar V. B.. (2012). Molecular analysis of gut microbiota in obesity among Indian individuals. J. Biosci. 37, 647–657. 10.1007/s12038-012-9244-0 22922190

[B60] Qin J. Li Y. Cai Z. Li S. Zhu J. Zhang F.. (2012). A metagenome-wide association study of gut microbiota in type 2 diabetes. Nature 490, 55–60. 10.1038/nature11450 23023125

[B61] Quast C. Pruesse E. Yilmaz P. Gerken J. Schweer T. Yarza P.. (2013). The SILVA ribosomal RNA gene database project: improved data processing and web-based tools. Nucleic Acids Res. 41, D590–D596. 10.1093/nar/gks1219 23193283 PMC3531112

[B62] R Core Team (2013). R: A Language and Environment for Statistical Computing. Vienna: R Foundation for Statistical Computing http://www.R-project.org/

[B63] Ramachandran A. Snehalatha C. Shetty A. S. Nanditha A. (2012). Trends in prevalence of diabetes in Asian countries. World J. Diabetes 3, 110–117. 10.4239/wjd.v3.i6.110 22737281 PMC3382707

[B64] Remely M. Aumueller E. Merold C. Dworzak S. Hippe B. Zanner J.. (2014). Effects of short chain fatty acid producing bacteria on epigenetic regulation of FFAR3 in type 2 diabetes and obesity. Gene 537, 85–92. 10.1016/j.gene.2013.11.081 24325907

[B65] Samuel B. S. Gordon J. I. (2006). A humanized gnotobiotic mouse model of host-archaeal-bacterial mutualism. Proc. Natl. Acad. Sci. U.S.A. 103, 10011–10016. 10.1073/pnas.0602187103 16782812 PMC1479766

[B66] Samuel B. S. Hansen E. E. Manchester J. K. Coutinho P. M. Henrissat B. Fulton R.. (2007). Genomic and metabolic adaptations of *Methanobrevibacter smithii* to the human gut. Proc. Natl. Acad. Sci. U.S.A. 104, 10643–10648. 10.1073/pnas.0704189104 17563350 PMC1890564

[B67] Scanlan P. D. Shanahan F. Marchesi J. R. (2008). Human methanogen diversity and incidence in healthy and diseased colonic groups using mcrA gene analysis. BMC Microbiol. 8:79. 10.1186/1471-2180-8-79 18492229 PMC2408590

[B68] Schloss P. D. Westcott S. L. Ryabin T. Hall J. R. Hartmann M. Hollister E. B.. (2009). Introducing mothur: open-source, platform-independent, community-supported software for describing and comparing microbial communities. Appl. Environ. Microbiol. 75, 7537–7541. 10.1128/AEM.01541-09 19801464 PMC2786419

[B69] Shannon C. E. (1948). A mathematical theory of communication. Bell Syst. Tech. J. 27, 379–423. 10.1002/j.1538-7305.1948.tb01338.x

[B70] Shannon P. Markiel A. Ozier O. Baliga N. S. Wang J. T. Ramage D.. (2003). Cytoscape: a software environment for integrated models of biomolecular interaction networks. Genome Res. 13, 2498–2504. 10.1101/gr.1239303 14597658 PMC403769

[B71] Soyucen E. Gulcan A. Aktuglu-Zeybek A. C. Onal H. Kiykim E. Aydin A. (2014). Differences in the gut microbiota of healthy children and those with type 1 diabetes. Pediatr. Int. 56, 336–343. 10.1111/ped.12243 24475780

[B72] Spor A. Koren O. Ley R. (2011). Unravelling the effects of the environment and host genotype on the gut microbiome. Nat. Rev. Microbiol. 9, 279–290. 10.1038/nrmicro2540 21407244

[B73] Suryavanshi M. V. Bhute S. S. Jadhav S. D. Bhatia M. S. Gune R. P. Shouche Y. S.. (2016). Hyperoxaluria leads to dysbiosis and drives selective enrichment of oxalate metabolizing bacterial species in recurrent kidney stone endures. Sci. Rep. 6:34712. 10.1038/srep34712 27708409 PMC5052600

[B74] Tang J. Iliev I. D. Brown J. Underhill D. M. Funari V. A. (2015). Mycobiome: approaches to analysis of intestinal fungi. J. Immunol. Methods 421, 112–121. 10.1016/j.jim.2015.04.004 25891793 PMC4451377

[B75] Teanpaisan R. Hintao J. Dahlén G. (2009). Oral Lactobacillus species in type 2 diabetic patients living in southern Thailand. Anaerobe 15, 160–163. 10.1016/j.anaerobe.2009.01.004 19655427

[B76] Turnbaugh P. J. Ley R. E. Mahowald M. A. Magrini V. Mardis E. R. Gordon J. I. (2006). An obesity-associated gut microbiome with increased capacity for energy harvest. Nature 444, 1027–1031. 10.1038/nature05414 17183312

[B77] Turnbaugh P. J. Ridaura V. K. Faith J. J. Rey F. E. Knight R. Gordon J. I. (2009). The effect of diet on the human gut microbiome: a metagenomic analysis in humanized gnotobiotic mice. Sci. Transl. Med. 1, 6ra14. 10.1126/scitranslmed.3000322 20368178 PMC2894525

[B78] Wang Z. K. Yang Y. S. Stefka A. T. Sun G. Peng L. H. (2014). Review article: fungal microbiota and digestive diseases. Aliment. Pharmacol. Ther. 39, 751–766. 10.1111/apt.12665 24612332

[B79] Yajnik C. S. (2001). The insulin resistance epidemic in India: fetal origins, later lifestyle, or both? Nutr. Rev. 59, 1–9. 10.1111/j.1753-4887.2001.tb01898.x 11281246

[B80] Yajnik C. S. (2004). Early life origins of insulin resistance and type 2 diabetes in India and other Asian countries. J. Nutr. 134, 205–210. 14704320 10.1093/jn/134.1.205

[B81] Yajnik C. S. Fall C. H. Vaidya U. Pandit A. N. Bavdekar A. Bhat D. S.. (1995). Fetal growth and glucose and insulin metabolism in four-year-old Indian children. Diabet. Med. 12, 330–336. 10.1111/j.1464-5491.1995.tb00487.x 7600749

[B82] Zhang X. Shen D. Fang Z. Jie Z. Qiu X. Zhang C.. (2013). Human gut microbiota changes reveal the progression of glucose intolerance. PLoS ONE 8:e71108. 10.1371/journal.pone.0071108 24013136 PMC3754967

