# Supplementary material for: Gut Microbial Diversity Assessment of Indian Type-2-Diabetics Reveals Alterations in Eubacteria, Archaea, and Eukaryotes
Source: Front Microbiol. 2017 Feb 14;8:214. doi: 10.3389/fmicb.2017.00214 (PMC5306211; doi:10.3389/fmicb.2017.00214)
Supplement: Supplementary file 1 [file Table1.DOC]

**Supplementary Table 1: Primer used during the PCR amplification of archaeal, eukaryotic and fungal communities.**

| Domain/Group | Name | Sequence (5’-3’) | Product Size | Reference |
| --- | --- | --- | --- | --- |
| Archaea | Archea806F | ATTAGATACCCSBGTAGTCC | 194 | (Gittel et al., 2009) |
|  | Archea1000R | GGCCATGCACYWCYTCTC |  | (Gantner et al., 2011) |
| Eukarya | Euk1209F | CAGGTCTGTGATGCCC | 183 | (Giovannoni et al., 1988) |
|  | Euk1392R | ACGGGCGGTGTGTRC |  | (Lane et al., 1985) |
| Fungal ITS | ITS1F | TCCGTAGGTGAACCTGCGG | Varied | (White et al., 1990) |
|  | ITS4R | TCCTCCGCTTATTGATATGC |  |  |

**References**

Gantner, S., Andersson, A. F., Alonso-Sáez, L., and Bertilsson, S. (2011). Novel primers for 16S rRNA-based archaeal community analyses in environmental samples. *J. Microbiol. Methods* 84, 12–8. doi:10.1016/j.mimet.2010.10.001.

Giovannoni, S. J., DeLong, E. F., Olsen, G. J., and Pace, N. R. (1988). Phylogenetic group-specific oligodeoxynucleotide probes for identification of single microbial cells. *J. Bacteriol.* 170, 720–726.

Gittel, A., Sorensen, K. B., Skovhus, T. L., Ingvorsen, K., and Schramm, A. (2009). Prokaryotic Community Structure and Sulfate Reducer Activity in Water from High-Temperature Oil Reservoirs with and without Nitrate Treatment. *Appl. Environ. Microbiol.* 75, 7086–7096.

Lane, D. J., Pace, B., Olsen, G. J., Stahl, D. A., Sogin, M. L., and Pace, N. R. (1985). Rapid determination of 16S ribosomal RNA sequences for phylogenetic analyses. *Proc. Natl. Acad. Sci.* 82, 6955–6959.

White, T., Bruns, T., Lee, S., and Taylor, J. (1990). "Amplification and direct sequencing of fungal ribosomal RNA genes for phylogenetics" in PCR Protocols: A Guide to Methods and Applications, ed. Ma Innis, Dh Gelfand, Jj Shinsky, Tj White, 315–322.
