# Supplementary material for: Gut Microbial Diversity Assessment of Indian Type-2-Diabetics Reveals Alterations in Eubacteria, Archaea, and Eukaryotes
Source: Front Microbiol. 2017 Feb 14;8:214. doi: 10.3389/fmicb.2017.00214 (PMC5306211; doi:10.3389/fmicb.2017.00214)

Supplementary Figure 1: Optimal number of clusters supporting the enterotypes as estimated using Calinski–Harabasz (CH) index: a) All Subjects, b) NGTs, c) New-DMs, and d) Known-DMs

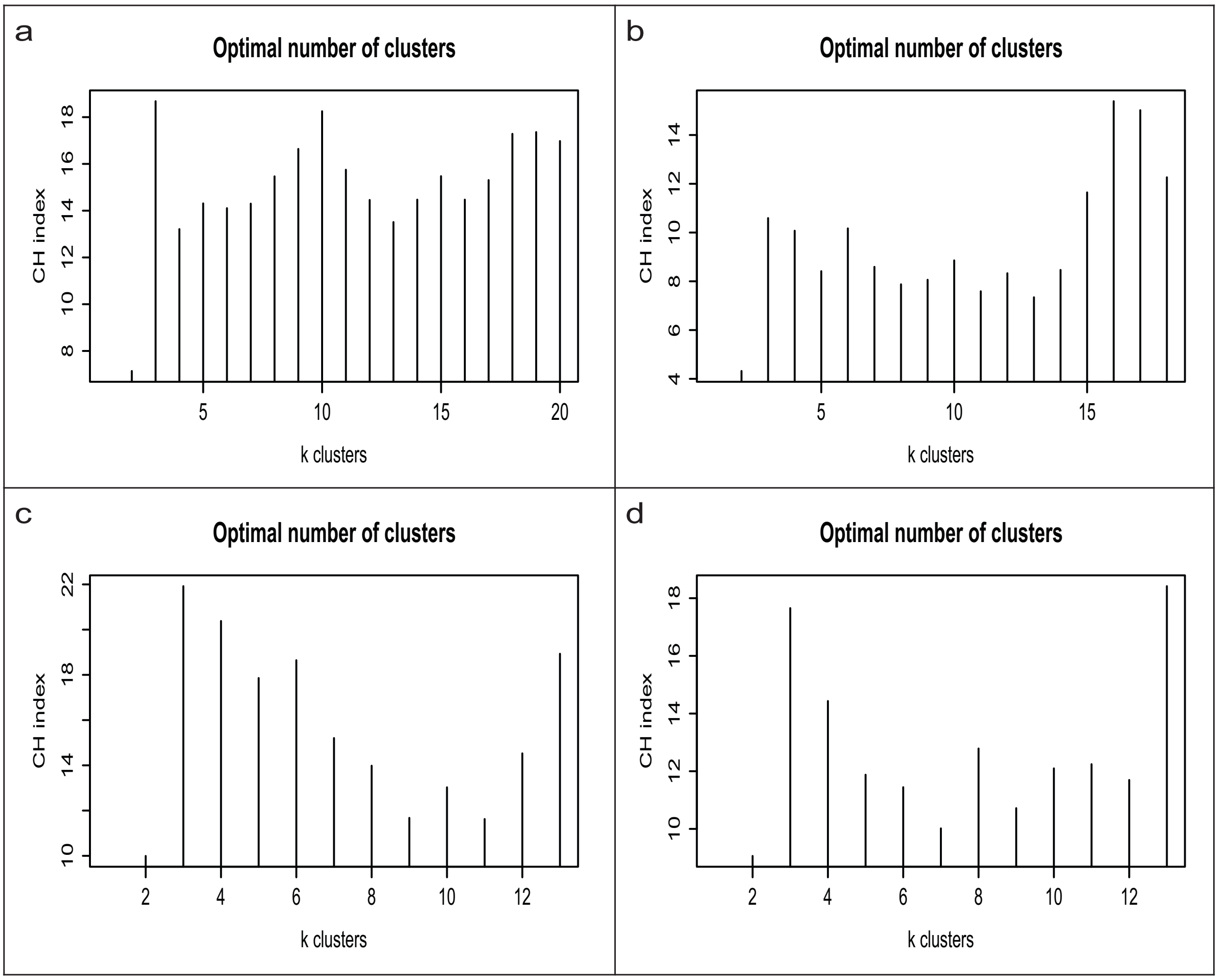

Supplement: Supplementary file 5 [file Image1.PDF]
