# Supplementary material for: Gut Microbial Diversity Assessment of Indian Type-2-Diabetics Reveals Alterations in Eubacteria, Archaea, and Eukaryotes
Source: Front Microbiol. 2017 Feb 14;8:214. doi: 10.3389/fmicb.2017.00214 (PMC5306211; doi:10.3389/fmicb.2017.00214)

Supplementary Figure 2: Node degree distributions of the network of co-occurrence, and co-exclusion associations. Node degree indicates the number of links that connect node to others in the network. Power law degree distributions means that most nodes have only a few edges and are often connected few high-degree hub nodes.

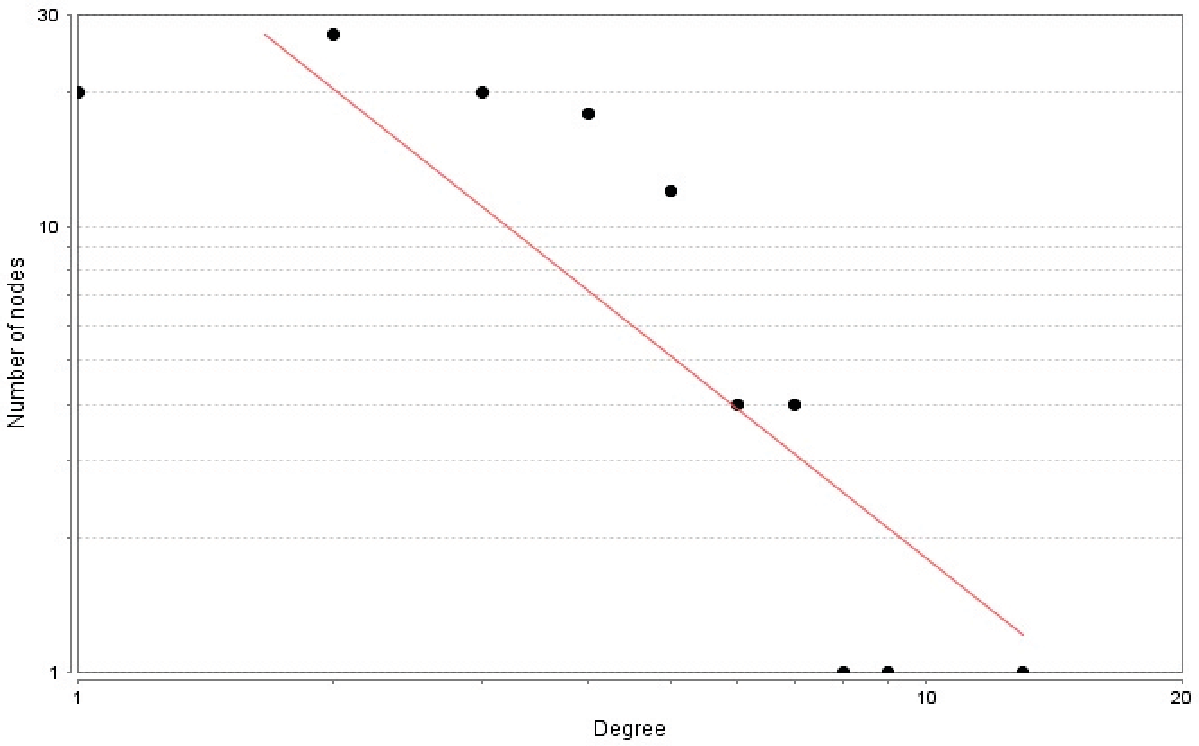

Supplement: Supplementary file 6 [file Image2.PDF]
