# Supplementary material for: Gut Microbial Diversity Assessment of Indian Type-2-Diabetics Reveals Alterations in Eubacteria, Archaea, and Eukaryotes
Source: Front Microbiol. 2017 Feb 14;8:214. doi: 10.3389/fmicb.2017.00214 (PMC5306211; doi:10.3389/fmicb.2017.00214)

Supplementary Figure 5: Significant co-occurrence relationships at genus level in Known-DMs subjects. Each node represents a bacterial genus; size of the node is proportional to the abundance of the genus. Each edge represents co-occurance relationships; edge width is proportional to the significance of supporting evidance.

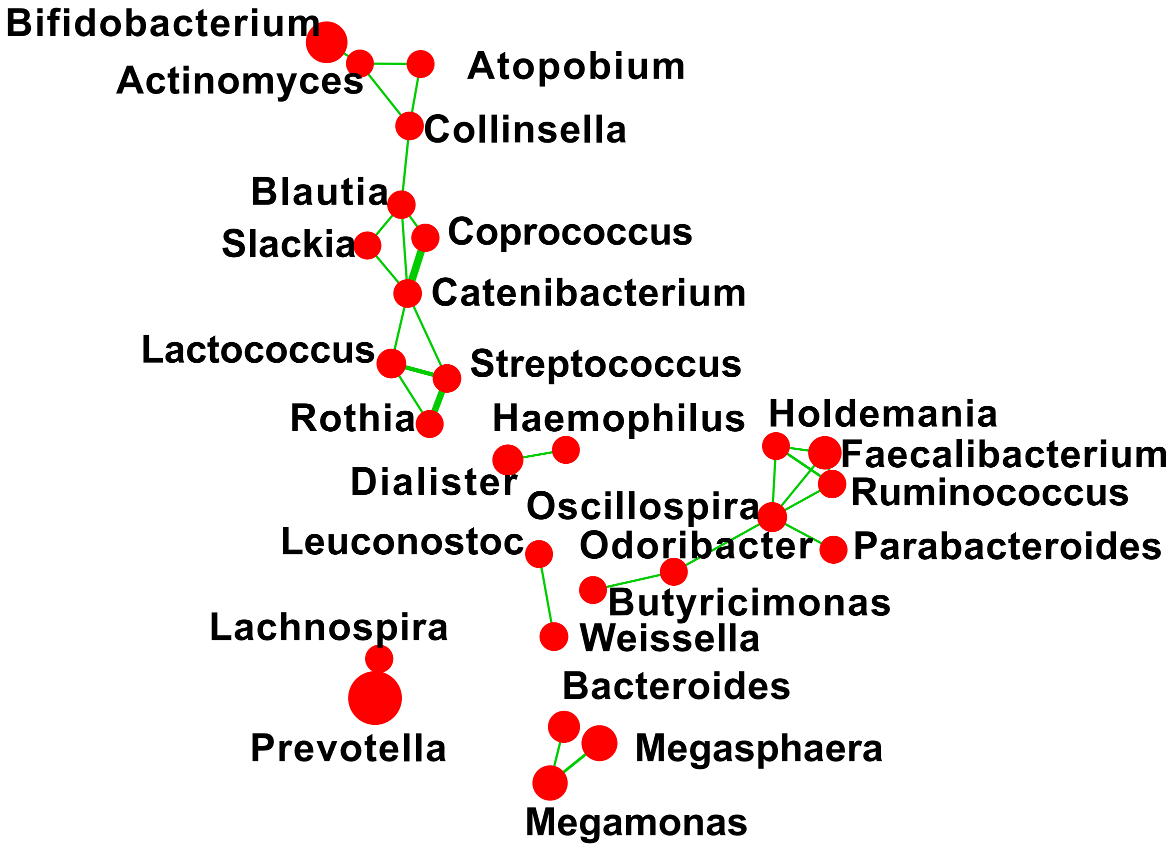

Supplement: Supplementary file 9 [file Image5.PDF]
