# Supplementary material for: Gut Microbial Diversity Assessment of Indian Type-2-Diabetics Reveals Alterations in Eubacteria, Archaea, and Eukaryotes
Source: Front Microbiol. 2017 Feb 14;8:214. doi: 10.3389/fmicb.2017.00214 (PMC5306211; doi:10.3389/fmicb.2017.00214)

Supplementary Figure 6: Extended error bar plot showing the differentially enriched KOs in NGTs subjects (green bars) as compared to New-DMs subjects (yellow bars)

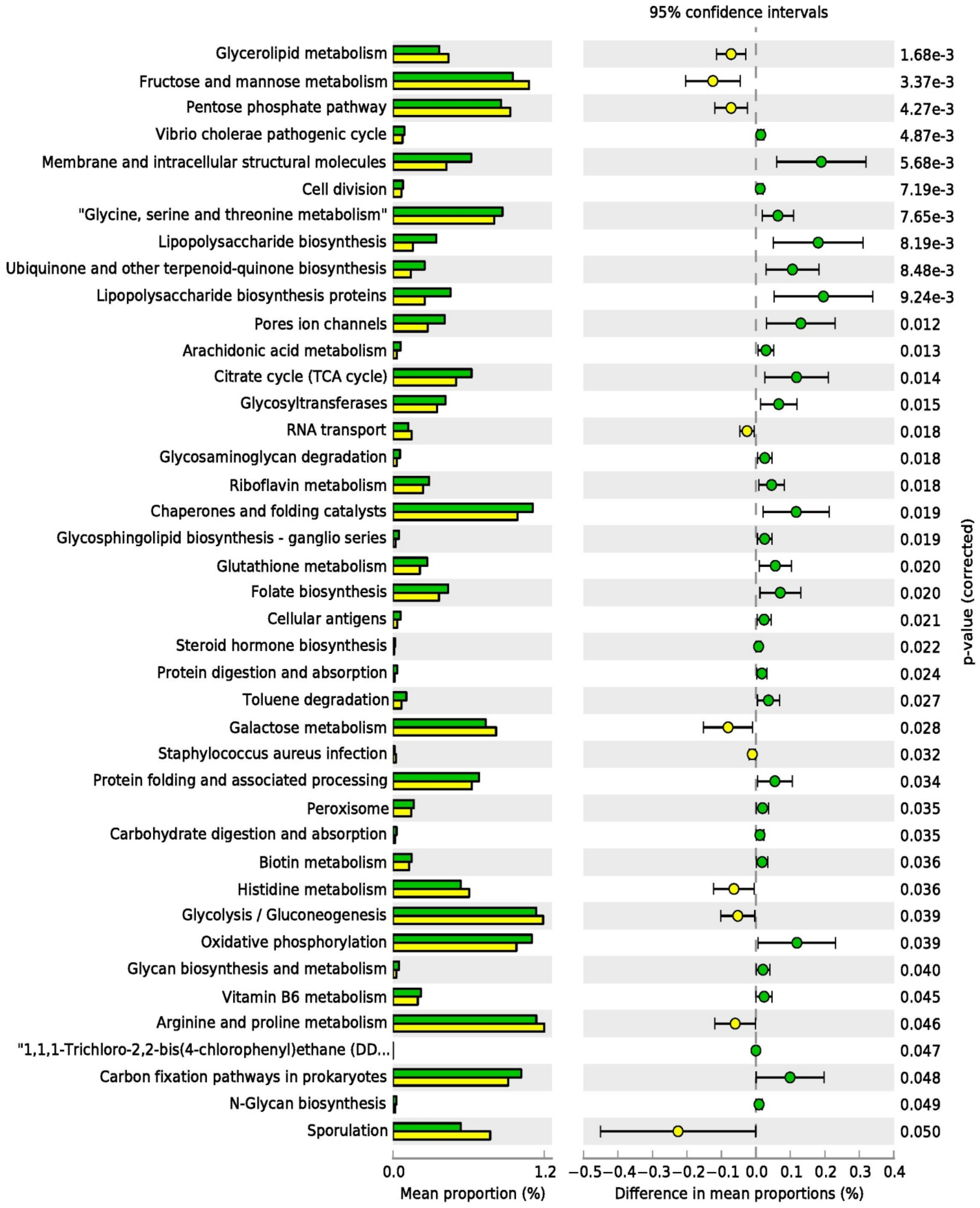

Supplement: Supplementary file 10 [file Image6.PDF]
